# Synchronous collaborative assessment using a comprehensive 5G telemedicine platform in otorhinolaryngology: Bridging the gap and enhancing health equity in rural areas through smart health care

**DOI:** 10.1371/journal.pdig.0001600

**Published:** 2026-08-04

**Authors:** You-Cheng Yu, Shih-Huan Chen, Yen-Che Wang, Sung-Huai Hsieh, Tang-Chuan Wang

**Affiliations:** 1 The PhD Program for Medical Engineering and Rehabilitation Science, College of Biomedical Engineering, China Medical University, Taichung City, Taiwan; 2 School of Medicine, College of Medicine, China Medical University, Taichung City, Taiwan; 3 Master Program for Digital Health Innovation, China Medical University, Taichung City, Taiwan; 4 Information Center, China Medical University Hsinchu Hospital, Zhubei City, Hsinchu County, Taiwan; 5 Department of Master Program for Biomedical Engineering, College of Biomedical Engineering, China Medical University, Taichung City, Taiwan; 6 Department of Otorhinolaryngology-Head and Neck Surgery, China Medical University Hsinchu Hospital, Zhubei City, Hsinchu County, Taiwan; Maastricht University Cardiovascular Research Institute Maastricht: Universiteit Maastricht Cardiovascular Research Institute Maastricht, NETHERLANDS, KINGDOM OF THE

## Introduction: The critical diagnostic gap in rural otorhinolaryngology

Telemedicine enhances healthcare accessibility by providing remote consultations, reducing geographical barriers, and improving timely access to medical services, ultimately leading to better patient outcomes and more cost-effective healthcare delivery [1]. However, significant disparities in healthcare access between rural and urban areas persist in the United States, where physician density remains consistently lower in rural areas across different healthcare systems (e.g., approximately 59.7 vs. 125.3 physicians per 100,000 population) [2]. Similar disparities have been reported in China, where urban areas have substantially higher physician density than rural areas [3]. This imbalance in healthcare workforce distribution restricts access to specialized services, elevates the diagnostic threshold, and contributes to delayed detection of complex pathologies. To expand telemedicine as a strategy to reduce these disparities, seamless interoperability among systems, user-friendly technology, reliable communication devices, and robust information security measures are essential [4,5]. Ensuring the security and upkeep of medical systems, as well as the development of standardized and intuitive user interfaces, is also paramount for delivering superior-quality healthcare services.

Otorhinolaryngology is a visually dependent specialty that relies heavily on high-resolution imaging and expert interpretation. The advancement of telemedicine in otorhinolaryngology is crucial as it can eliminate the need for in-person visits and extend specialized care to underserved areas, a need that became particularly evident during the COVID-19 pandemic [1,6]. However, several challenges persist in developing telemedicine, especially in rural areas [7,8]. Specifically, the effectiveness of telemedicine in otorhinolaryngology is fundamentally constrained by underlying communication infrastructure, which is often less developed in rural settings. Rural areas often still experience heterogeneous 5G coverage, limited fiber backhaul availability, and continued reliance on 4G/LTE networks, which constrain the full deployment of advanced 5G-enabled services. Furthermore, the feasibility of deploying 5G Network Slicing in rural healthcare environments is constrained by substantial infrastructure and operational costs, as well as increased system complexity associated with network function virtualization and edge-based orchestration [9,10]. Prior studies have also highlighted that rural 5G deployment is often economically challenging due to low population density and high capital expenditure requirements per user [11]. These factors collectively limit adoption by smaller rural healthcare providers with constrained financial and technical resources.

These infrastructure limitations ultimately translate into clinical constraints in rural otorhinolaryngology, where diagnostic accuracy depends heavily on high-resolution visual interpretation. The absence of immediate specialist input during critical diagnostic windows creates a “diagnostic dead zone,” which results in rural patients facing a significantly higher likelihood of advanced-stage diagnosis compared to urban populations. Population-based studies of head and neck malignancies, including oral cancer, have reported up to a 35% increased odds of late-stage presentation in rural populations [12]. Otorhinolaryngology diagnosis relies heavily on endoscopic and high-resolution visual assessment due to subtle mucosal changes that require expert interpretation in real time. Therefore, high-fidelity, low-latency communication is essential for supporting timely clinical decision-making and reducing the risk of delayed diagnosis in rural settings.

This paper is not intended as a technical report of a specific platform but rather reports this implementation as an illustrative case to critically examine how current healthcare systems fail to support timely diagnosis in rural settings, as well as how 5G-enabled synchronous collaboration can enable a transition toward network-centric, distributed models of care.

## Technical framework: Beyond bandwidth to synchronous collaboration

The objective of this report is to describe a collaborative synchronous telemedicine assessment platform to aid physicians in jointly diagnosing these relatively complex disorders. While previous telemedicine approaches were limited by bandwidth constraints and transmission lag, remote consultation via a 5G platform using advanced medical imaging (e.g., endoscopy) may accurately identify crucial clinical findings. This platform features 5G synchronization, fast transmission, low latency, and high bandwidth (e.g., transmitting a 3 GB ultrasound image takes only 34.3 seconds, compared to 240 seconds with 4G). It integrates with both ends of the Picture Archiving and Communication System (PACS) and Hospital Information System (HIS), offering a video interface for real-time patient observation. However, real-world interoperability between HIS and PACS remained challenging due to heterogeneous data standards, vendor-specific implementations, and the lack of seamless real-time synchronization across institutional tiers. To address these barriers, we adopted a structured Enterprise Architecture based on HL7 Fast Healthcare Interoperability Resources (FHIR) and HL7 interface engine (broker) mechanisms. This has enabled standardized data exchange and reduced manual data entry across systems, which has been reported to improve clinical workflow efficiency and shorten report turnaround time [13–15].

**Fig 1:**
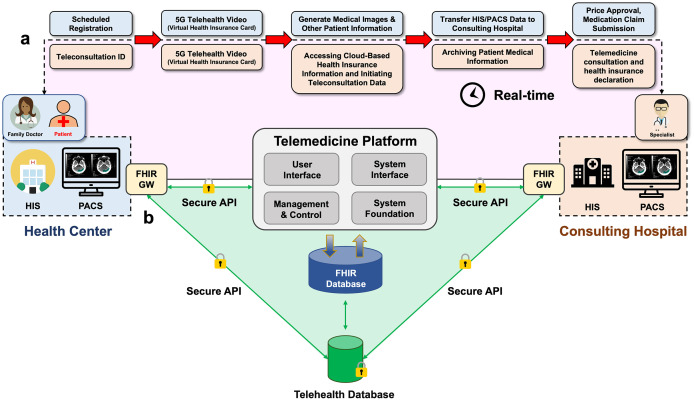
Overview of the 5G telemedicine platform and real-world clinical workflow. **(a)** Schematic representation of the end-to-end telemedicine consultation process, including patient registration, real-time synchronous interaction between local physicians and remote specialists during endoscopic examination, diagnostic support, and subsequent administrative and reporting procedures. **(b)** System architecture of the telemedicine platform, illustrating secure data transmission between health centers and consulting hospitals via FHIR Gateway, HIS, and PACS integration, supported by encrypted APIs and centralized FHIR/telehealth databases for patient information management and interoperability. This figure is intended to bridge clinical workflow and system architecture, illustrating how synchronous telemedicine is enabled through integrated communication and data infrastructure.

Fig 1a illustrates clinical practice using the telemedicine platform, and Fig 1b shows patient data security management. The platform employs 5G Network Slicing to prioritize medical traffic as follows: (a) Ultra-Reliable Low-Latency Communications (uRLLC) Slice: Dedicated to the control plane, achieving <10 ms latency to ensure “Virtual Proctoring” commands and verbal instructions remain synchronized with the physical examination. (b) Enhanced Mobile Broadband (eMBB) Slice: Provides a sustained >500 Mbps throughput, supporting uncompressed 4K HDR endoscopic video without compression artifacts.

## Clinical validation: A case for synchronous intervention

Compared to the traditional “store-and-forward” model—where asynchronous review of medical images is associated with significant diagnostic delays and limited interactive feedback—this 5G synchronous platform enables real-time navigation and immediate clinical decision-making. Prior studies have reported that store-and-forward telemedicine systems can experience delays exceeding 12 hours in over 10% of cases, with some cases delayed by several days, leading to postponed diagnosis and intervention. In contrast, real-time telemedicine facilitates direct interaction and reduces the need for follow-up consultations, thereby significantly shortening the time to diagnosis from hours or days to minutes [16,17].

Nasal obstruction can be clinically challenging, especially for rare disorders, because of a wide differential diagnosis. For example, our institution evaluated a 45-year-old man with intermittent nasal congestion for 10 years that had progressively worsened and become persistent in the past 3 months. At a local medical facility, the physician used a headlight-mounted video camera for remote consultation with an experienced otorhinolaryngologist at our hospital. Real-time images were visible at both facilities through the use of a 5G telemedicine platform. Flexible endoscopy was performed to check the lesion, and the remote specialist guided the onsite physician to navigate toward the olfactory cleft, facilitating early diagnosis and prompt referral of the patient with olfactory neuroblastoma.After surgery, the patient was monitored via the telemedicine system for 5 years, showing stable clinical outcomes and no recurrence. Olfactory neuroblastoma, an uncommon malignant neuroectodermal tumor originating from the upper nasal cavity and/or skull base, represents 2% of all sinonasal tract tumors [18] and occurs over a wide age range (3–90 years) [19]. Patients with Olfactory neuroblastoma commonly present with unilateral nasal obstruction (93%) and epistaxis (55%) [19]. The lesions can be easily missed due to symptoms that mimic nasal polyps, chronic rhinosinusitis, or benign nasal tumors [20,21]. Moreover, early symptoms are non-specific, which leads to an average delay of 6–12 months from the onset of symptoms until diagnosis [22]. This case illustrates how 5G-enabled telemedicine may facilitate timely specialist involvement in diagnostically challenging cases occurring in resource-limited settings. While conclusions regarding diagnostic accuracy and long-term outcomes cannot be drawn from a single case, the experience highlights the potential value of synchronous collaborative assessment in supporting earlier recognition and referral of rare otolaryngologic diseases.

## Discussion: The future of digital health and artificial intelligence (AI)

Besides focusing on a single technological solution, this study highlights a broader system-level challenge: the mismatch between centralized specialist expertise and the distributed nature of patient populations in rural settings. By addressing this gap through a synchronous, network-enabled framework, specialist care can be more consistently extended beyond tertiary centers and integrated into routine care pathways in underserved regions.

(a) **Addressing Global Disparities in Healthcare Access Based on 5G-powered Diagnostic Hub:** The global disparity in healthcare access between urban and rural regions remains a persistent challenge, necessitating a shift from traditional outreach to high-capacity digital interventions. In Canada, disparities in healthcare access are evident, with socioeconomic status, racialization, and place of residence (e.g., rural vs. urban) significantly influencing access to primary and specialized care [23]. Similarly, Alaska faces a shortage of healthcare professionals, particularly in rural areas, necessitating long journeys for specialty care [24]. India’s rural population also experiences a high prevalence of otorhinolaryngology disorders, underscoring the need for improved healthcare access [25]. In New South Wales and the Australian Capital Territory, limited outreach initiatives leave many remote communities without adequate healthcare services [26]. These examples illustrate the global challenge of unequal healthcare access and the importance of innovative solutions like telemedicine for bridging the gap. By transforming the rural clinic from a mere referral point into a 5G-powered diagnostic hub, linking to corresponding resources, this platform represents a potentially scalable framework that may help address healthcare access disparities, although its effectiveness, scalability, and cost-effectiveness require validation through larger real-world implementation studies.
(b) **Strategic Evolution,From Connectivity to Intelligence:** To move beyond basic connectivity, the high-resolution imaging data generated by this 5G platform must be recognized as a critical medical asset. Managing these digital health assets requires a framework that balances data security with multi-tier interoperability, using protocols such as HL7 FHIR implemented in this study, to bridge PACS and HIS and ensure that high-fidelity data remain accessible across the clinical continuum. However, the effective utilization of such data remains challenging due to heterogeneity in imaging quality, variability across acquisition devices, and limited availability of well-annotated datasets for supervised learning. In addition, concerns related to data governance, privacy, and cross-institutional data sharing further complicate large-scale AI deployment [27–29]. To address these challenges, the integration of multimodal AI provides a promising pathway toward scalable and intelligent diagnostics. Recent advances in deep learning and generative AI enable the analysis of complex medical imaging and real-time video streams, facilitating automated decision support and “second-opinion” systems. Emerging strategies such as federated learning, edge-based inference, and multimodal data fusion are of particular interest in distributed telemedicine settings, allowing for model training and deployment across institutions without compromising data privacy. By synergizing 5G-enabled high-throughput transmission with AI-driven diagnostic support, this platform has the potential to reduce cognitive burden on frontline clinicians, improve diagnostic efficiency, and enhance access to specialized care in resource-limited regions [30–32]. However, these anticipated benefits remain largely hypothesis-generating and will require prospective clinical validation before widespread adoption can be recommended. This convergence of connectivity and intelligence represents a critical step toward achieving scalable and equitable global healthcare systems.

(c) **Telemedicine in Otolaryngology, driving Decentralization on Health Care:** The clinical implications of this framework are particularly evident in rural otolaryngology practice. In Alaska, store-and-forward telemedicine effectively complemented otolaryngology services by allowing for asynchronous communication. This method, convenient for both sender and receiver, optimizes clinical time by enabling data collection from the patient before consultation [24]. Similarly, in Tamil Nadu, India, community workers were trained to use video otoscopy for remote diagnostic evaluations, including synchronous pure tone audiometry and tympanometry for middle ear diseases. During the COVID-19 pandemic, telemedicine services, utilizing InTouch Lite technology, were implemented to facilitate real-time physician-patient interaction in Taiwan. Otolaryngology healthcare is a crucial service that rural areas often lack. Therefore, establishing a disease triage system is imperative. Our solution utilizes a closed-loop care model, and the 5G link transforms the rural clinic from a simple triage site into a specialized diagnostic outpost. This decentralization of expertise may help optimize the clinical pathway, reserving tertiary resources for surgical intervention while shifting long-term surveillance back to the community level via 5G-enabled “virtual clinics. This system may facilitate more targeted referral decisions and reduce delays associated with prolonged observation, although comparative effectiveness studies are still needed. Beyond clinical workflow optimization, this framework also reflects a broader paradigm shift toward the decentralization of medical expertise, where diagnostic authority is progressively redistributed from tertiary centers to peripheral healthcare settings. Such a transition challenges the traditional hospital-centric model and underscores the need for corresponding adjustments in healthcare policy and reimbursement structures to support network-centric care delivery. Recent telemedicine scalability studies highlighted that workable telemedicine reimbursement is a critical enabling factor in expanding health care access. [33].

## Study limitations

Several limitations should be acknowledged. First, an illustrative clinical case was presented rather than a prospective outcome study in this article. Therefore, the observations should be interpreted as demonstrating the feasibility and potential clinical utility of synchronous 5G-enabled telemedicine rather than providing definitive evidence of improved patient outcomes. Second, the objective of this report was not to directly compare different telemedicine models or referral pathways, but rather to illustrate how real-time specialist collaboration may facilitate diagnostic decision-making in resource-limited settings. Third, implementation of such systems will likely require adaptation according to local network infrastructure, healthcare resources, and reimbursement frameworks. Nevertheless, the present case provides a practical example of how advanced communication technologies can support distributed specialist care and serves as a foundation for future studies evaluating clinical effectiveness, scalability, and health-system impact.

## Conclusion

In conclusion, the integration of advanced imaging technologies and real-time collaboration between specialists highlights the role of telemedicine in bridging healthcare disparities, particularly in underserved areas. By addressing technical, organizational, and security challenges, 5G platforms may improve diagnostic workflows and facilitate timely specialist involvement, particularly in geographically underserved settings.

More importantly, this work proposes a potential transition from a hospital-centric model toward a network-centric model of care delivery, enabling the decentralization of medical expertise to peripheral healthcare settings. Achieving this transformation requires not only technological advancement but also a corresponding change in healthcare policy and reimbursement frameworks that support sustainable and scalable implementation.

## References

[1] Moentmann MR, Miller RJ, Chung MT, Yoo GH. Using telemedicine to facilitate social distancing in otolaryngology: a systematic review. J Telemed Telecare. 2023;29(5):331–48. doi: 10.1177/1357633X20985391 33535916

[2] Basu S, et al. Association of primary care physician supply with population mortality in the United States, 2005-2015. JAMA Intern Med. 2019;179(4):506–14. 30776056 10.1001/jamainternmed.2018.7624 PMC6450307

[3] Xiao Y, Zhang Z, Xu C-M, Yu J-Y, Chen T-T, Jia S-W, et al. Evolution of physician resources in China (2003-2021): quantity, quality, structure, and geographic distribution. Hum Resour Health. 2025;23(1):15. doi: 10.1186/s12960-025-00983-8 40055804 PMC11889850

[4] Garg V, Brewer J. Telemedicine security: a systematic review. J Diabetes Sci Technol. 2011;5(3):768–77. doi: 10.1177/193229681100500331 21722592 PMC3192643

[5] Klaassen B, van Beijnum BJF, Hermens HJ. Usability in telemedicine systems: a literature survey. Int J Med Inform. 2016;93:57–69. doi: 10.1016/j.ijmedinf.2016.06.004 27435948

[6] Fieux M, Duret S, Bawazeer N, Denoix L, Zaouche S, Tringali S. Telemedicine for ENT: effect on quality of care during Covid-19 pandemic. Eur Ann Otorhinolaryngol Head Neck Dis. 2020;137(4):257–61. doi: 10.1016/j.anorl.2020.06.014 32624390 PMC7306717

[7] Bali S. Barriers to development of telemedicine in developing countries. In Telehealth. IntechOpen; 2018.

[8] Mousavi Baigi SF, Mousavi Baigi SM, Mazaheri Habibi MR. Challenges and opportunities of using telemedicine during covid-19 epidemic: a systematic review. Front Health Inform. 2022;11(1):109. doi: 10.30699/fhi.v11i1.346

[9] Ferreira JP, Ferreira VC, Nogueira SL, Faria JM, Afonso JA. A flexible infrastructure-sharing 5G network architecture based on network slicing and roaming. Information. 2024;15(4):213. doi: 10.3390/info15040213

[10] Walia JS, Hämmäinen H, Kilkki K, Flinck H, Yrjölä S, Matinmikko-Blue M. A virtualization infrastructure cost model for 5G network slice provisioning in a smart factory. JSAN. 2021;10(3):51. doi: 10.3390/jsan10030051

[11] Maluleke H, Bagula A, Ajayi O, Chiaraviglio L. An economic feasibility model for sustainable 5G networks in rural dwellings of South Africa. Sustainability. 2022;14(19):12153. doi: 10.3390/su141912153

[12] Tsai E, Walker B, Wu S-C. Nationwide oral cancer screening and rural-urban disparities in oral cancer diagnosis, treatment and mortality: a population-based cohort study in Taiwan. BMJ Public Health. 2026;4(1):e003963. doi: 10.1136/bmjph-2025-003963 41736809 PMC12927324

[13] Bender D, Sartipi K. HL7 FHIR: An Agile and RESTful approach to healthcare information exchange. In: Proceedings of the 26th IEEE International Symposium on Computer-Based Medical Systems, 2013. p. 326–31. 10.1109/cbms.2013.6627810

[14] Popov M, Mihanovic F, HL7 functionalities in RIS/PACS/HIS system integration. Radiol J. 2022;46(1):12–8.

[15] Andriole KP. Picture archiving and communication systems: past, present, and future. J Med Imaging (Bellingham). 2023;10(6):061405. doi: 10.1117/1.JMI.10.6.061405 38162316 PMC10754358

[16] Gang Wang, Shan Lin, Mullen-Fortino M, Sokolsky O, Insup Lee. Transmission delay performance in telemedicine: a case study. Annu Int Conf IEEE Eng Med Biol Soc. 2017;2017:3723–7. doi: 10.1109/EMBC.2017.8037666 29060707

[17] Loane MA, Bloomer SE, Corbett R, Eedy DJ, Hicks N, Lotery HE, et al. A randomized controlled trial to assess the clinical effectiveness of both realtime and store-and-forward teledermatology compared with conventional care. J Telemed Telecare. 2000;6 Suppl 1:S1-3. doi: 10.1258/1357633001933952 10793956

[18] Thompson LDR. Olfactory neuroblastoma. Head Neck Pathol. 2009;3(3):252–9. doi: 10.1007/s12105-009-0125-2 20596981 PMC2811627

[19] Lund VJ, et al. Olfactory neuroblastoma: past, present, and future? Laryngoscope. 2003;113(3):502–7. 12616204 10.1097/00005537-200303000-00020

[20] Argiris A, Dutra J, Tseke P, Haines K. Esthesioneuroblastoma: the Northwestern University experience. Laryngoscope. 2003;113(1):155–60. doi: 10.1097/00005537-200301000-00029 12514401

[21] Orlandi RR, et al. International consensus statement on allergy and rhinology: rhinosinusitis 2021. In: International forum of allergy & rhinology. Wiley Online Library; 2021. 10.1002/alr.22741 33236525

[22] Faragalla H, Weinreb I. Olfactory neuroblastoma: a review and update. Adv Anat Pathol. 2009;16(5):322–31. doi: 10.1097/PAP.0b013e3181b544cf 19700942

[23] Lavergne MR, et al. *Disparities in access to primary care are growing wider in Canada*. In Healthcare Management Forum. Los Angeles, CA: SAGE Publications; 2023. 10.1177/08404704231183599 PMC10447912 37340726

[24] Kokesh J, Ferguson AS, Patricoski C. The Alaska experience using store-and-forward telemedicine for ENT care in Alaska. Otolaryngol Clin North Am. 2011;44(6):1359–74, ix. doi: 10.1016/j.otc.2011.08.010 22032488

[25] Singh A, Kumar S. A survey of ear, nose and throat disorders in rural India. Indian J Otolaryngol Head Neck Surg. 2010;62(2):121–4. doi: 10.1007/s12070-010-0027-3 23120697 PMC3450296

[26] Shein G, Hunter K, Gwynne K, Tobin S, Kong K, Lincoln M, et al. The O.P.E.N. Survey: outreach projects in Ear, Nose and Throat (ENT) in New South Wales. Aust J Otolaryngol. 2019;2:0–0. doi: 10.21037/ajo.2019.04.01

[27] Topol EJ. High-performance medicine: the convergence of human and artificial intelligence. Nat Med. 2019;25(1):44–56. doi: 10.1038/s41591-018-0300-7 30617339

[28] Kahn MG, Callahan TJ, Barnard J, Bauck AE, Brown J, Davidson BN, et al. A harmonized data quality assessment terminology and framework for the secondary use of electronic health record data. EGEMS (Wash DC). 2016;4(1):1244. doi: 10.13063/2327-9214.1244 27713905 PMC5051581

[29] Kelly CJ, Karthikesalingam A, Suleyman M, Corrado G, King D. Key challenges for delivering clinical impact with artificial intelligence. BMC Med. 2019;17(1):195. doi: 10.1186/s12916-019-1426-2 31665002 PMC6821018

[30] Wang J, Lin A, Huang Y, Li G, Chen T, Sun C, et al. Medical data as a key asset in the digital health era: a framework for challenges and strategies. iMetaMed. 2025;1(2). doi: 10.1002/imm3.70014

[31] Lv J, Slowik A, Rani S, Kim B-G, Chen C-M, Kumari S, et al. Multimodal metaverse healthcare: a collaborative representation and adaptive fusion approach for generative artificial-intelligence-driven diagnosis. Research (Wash D C). 2025;8:0616. doi: 10.34133/research.0616 40078668 PMC11899152

[32] Liu Z, Xu J, Yin C, Han G, Che Y, Fan G, et al. Development and external validation of an artificial intelligence-based method for scalable chest radiograph diagnosis: a multi-country cross-sectional study. Research (Wash D C). 2024;7:0426. doi: 10.34133/research.0426 39109248 PMC11301699

[33] Huang-Ku E, Muenkaew P, Chavarina KK, Tun YM, Win ZN, Isaranuwatchai W, et al. Telemedicine public reimbursement models for national and subnational jurisdictions: scoping review. J Med Internet Res. 2025;27:e75478. doi: 10.2196/75478 40794105 PMC12341443

